# Induction of Cross-Reactive and Protective Antibody Responses After DNA Vaccination With MHCII-Targeted Stem Domain From Influenza Hemagglutinin

**DOI:** 10.3389/fimmu.2020.00431

**Published:** 2020-03-25

**Authors:** Gunnveig Grødeland, Marta Baranowska-Hustad, Justin Abadejos, Tanya R. Blane, John Teijaro, David Nemazee, Bjarne Bogen

**Affiliations:** ^1^K.G. Jebsen Centre for Influenza Vaccine Research, Institute of Clinical Medicine, University of Oslo and Oslo University Hospital, Oslo, Norway; ^2^Department of Immunology and Microbiology, The Scripps Research Institute, San Diego, CA, United States

**Keywords:** DNA vaccine, hemagglutinin (HA), MHCII, APC-targeted antigen, stem domain, VH1-69

## Abstract

Novel and more broadly protective vaccines against influenza are needed to efficiently meet antigenic drift and shift. Relevant to this end, the stem domain of hemagglutinin (HA) is highly conserved, and antibodies specific for epitopes located to the stem have been demonstrated to be able to confer broad protection against various influenza subtypes. However, a remaining challenge is to induce antibodies against the poorly immunogenic stem by vaccination strategies that can be scaled up for prophylactic vaccination of the general population. Here, we have developed DNA vaccines where the conserved stem domain of HA from influenza A/PR/8/34 (H1N1) and A/Shanghai/2/2013 (H7N9) was targeted toward MHC class II molecules on antigen-presenting cells (APC) for increased immunogenicity. Each of these vaccines induced antibodies that cross-reacted with other subtypes in the corresponding phylogenetic influenza groups. Importantly, when mixing the MHCII-targeted stem domains from H1N1 and H7N9 influenza viruses into one vaccine bolus, we observed broad protection against candidate stains from both phylogenetic groups 1 and 2.

## Introduction

Conventional influenza vaccines cannot provide protection against antigenically drifted or shifted strains of influenza viruses. Thus, novel vaccines that induce broadly protective immune responses are needed. Such vaccines will be of value both to rapidly counter an emerging pandemic strain, and during years where the conventional influenza vaccines fail to match circulating viruses (1).

The most abundantly expressed surface protein on influenza is hemagglutinin (HA), and its globular head represents the prime target for neutralizing antibodies that confer sterilizing immunity against specific strains of influenza. However, the immunodominant head domain rapidly acquires expressed mutations (antigenic drift) that cause escape from pre-existing immunity. In contrast, the stem structure connecting the globular head with the viral membrane is highly conserved between different influenza subtypes (2, 3). In line with this, stem-specific monoclonal antibodies (mAbs) can cross-react between different subtypes of influenza (4, 5) and mediate cross-protection (6–9).

The broadly protective potential of stem-specific mAbs has instigated several attempts to induce similar polyclonal antibodies by vaccination. A headless HA stem domain was constructed already in 1983 (10), but the acid treatment used to remove the HA1 subunit most likely also disrupted the epitopes to which broadly cross-reactive antibodies could be raised. As biotechnology developed, antigens could be engineered with higher precision. Headless HA containing the whole of HA2 and parts of HA1 could be incorporated into virus-like particles (VLPs) (11), or recombinant proteins constructed with region-specific stabilizing elements and fusions to trimerization domains derived from the leucine zipper or HIV gp41 (12, 13). Several groups have also used protein minimization to achieve a stabile HA stem structure (14–16), whereas others have engineered sequences for cell-free synthesis (17), or insect cell production (18), to express functional HA stem antigens. The common theme is delivery of a recombinant protein where the stem has been stabilized for structure and trimerization.

Here, we have taken a different approach. In order to enhance immunogenicity, the HA stem was inserted into a previously described vaccine format where antigens are targeted to antigen-presenting cells (APC) (19–21). The APC-targeted HA stem domain was encoded as a single gene in vaccine plasmids to allow for rapid upscaling and production and delivered as naked DNA. Following plasmid delivery, the DNA is taken up by cells at the injection site. Next, transfected cells secrete DNA-encoded vaccine proteins that target APC for delivery of antigen. Depending on the surface molecule that is targeted and the APC, immune responses can be polarized to different types of immunity (22, 23). To obtain predominantly antibody responses, we here chose to target the HA stem to major histocompatibility class II (MHCII) molecules. We found that MHCII-targeted DNA delivery of stem HA enhanced induction of cross-reactive antibodies and conferred antibody-mediated protection against lethal challenges with influenza.

## Materials and Methods

### Mice and Cell Lines

Six- to eight-week-old female BALB/c mice were purchased from Taconic (Ry, Denmark) and housed under minimal disease conditions. All the experiments with BALB/c were approved by the Norwegian Animal Research Authority (NARA). F1 mice of BALB/c and knock-in CR9114 gH mice (C57BL/6 background) were bred and included in experiments from a minimal age of 8 weeks. All experiments with the F1 mice were approved by the Institutional Animal Care and Use Committee at The Scripps Research Institute, and were performed in accordance with relevant institutional and national guidelines. CR9114 gH mice will be described in detail elsewhere (T. Ota, D. Nemazee); briefly, they were generated as previously described (24), except carrying a germline-reverted version of the VDJh element of CR9114 (25), encoding the amino acid sequence QVQLVQSGAEVKKPGSSVKVSCKASGGTFSSYAISWVRQAPGQGLEWMGGIIPIFGTANYAQKFQGRVTITADESTSTAYMELSSLRSEDTAVYYCARHGNYYYYSGMDVWGKGTTVTV. The HEK293E cell line was purchased from ATCC (Manassas, VA, USA).

### Molecular Cloning of Vaccine Constructs

In order to construct vaccines encoding the HA stem domain from influenza A/PR/8/1934 (H1N1), primers were equipped with SfiI-sites (underlined) and designed to pick up amino acids 18–60 and 292–532 (numbering according to H0): primer set 18–60: GGCCTCGGTGGCCTGGACACAATATGTATAGGCand TCCTCCTCCTCCACATAGTTTTCCGTTG; primer set 292–532: GGAGGAGGAGGATGTAACACGAAGTGand ACCGGCCCTGCAGGCCTCACGCCAGAATCTGATAGATCCCC. The fragments were joined by PCR SOEing with the linker: KLCGGGGCNTK(11). In order to construct vaccines encoding the HA stem domain from influenza A/Shanghai/2/2013 (H7N9), the gene segments encoding aa 19–61 and 289–535 (joined by KLCGGGGCNTK) were ordered from GenScript with flanking SfiI-sites as above. Next, the different stem domains were inserted by subcloning into the previously used APC-targeted vaccine format (20, 23), and the vaccine molecules inserted into pUMVC expression vectors (26) (kind gift from Bob Weinberg, Addgene plasmid #8449). In addition to the vaccines encoding the HA stem domains and a single-chain variable fragment (scFv) specific for MHC II molecules (I-E^d^) as targeting unit, or with the chemokine macrophage inflammatory protein 1 alpha (MIP1α) as targeting unit, a vaccine where the targeting unit had been replaced with a scFv specific for the hapten 4-hydroxy-3-iodo-5-nitrophenylacetic acid (NIP) (non-targeted control) (20, 23) was constructed with the stem domain from H1N1 influenza. In order to allow a comparison of responses to the full-length HA, the previously described αMHCII-HA (aa 18–541 of HA) was also used as a control (20).

### ELISA for Detection of Vaccine Proteins

Ninety-six-well plates (Costar 3590) were coated with mouse anti-human IgG (against C_H_3 domain) (MCA878G, 1 μg/ml, AbD serotec, Oxford, UK) or NIP-BSA. Plates were blocked with 0.1% BSA in PBS, and supernatants from HEK293E cells were transiently transfected with 1 μg of vaccine plasmids, or purified vaccine proteins were added to wells in triplicates. Plates were now incubated with biotinylated mAb against IgG (Fc fragment) (HP-6017, 1 μg/ml, Sigma-Aldrich, Germany) or a C179 mAb specific for stem HA (1 μg/ml, kind gift from Yoshinobu Okuno, Osaka University, Japan) followed by biotinylated anti mouse IgG2a [1 μg/ml IgG2a(a) 553502, BD Pharmingen]. Next, plates were incubated with streptavidin alkaline phosphatase (1:3000, GE Healthcare, USA), developed with phosphatase substrate (P4744-10G, Sigma-Aldrich, Germany), and read at 405 nm with a Tecan reader using the Magellan v5.03 program.

### ELISA for Detection of Serum Antibodies

Blood was harvested by puncture of the saphenous vein, and sera were collected by centrifugation. Ninety-six-well plates (Costar 3590) were coated with inactivated A/PR/8/34 (H1N1) (PR8) virus (1:1600) (virus supplied from Charles River, USA, with HA titer 1:65,536 in 0.05 ml) in PBS azide, or with recombinant HA protein (1 μg/ml) from one of the following viruses: PR8 (11684-V08H, Sino Biological Inc., USA), A/Hong Kong/1/1968 (H3N2) (40116-V08H1, Sino Biological Inc.), A/Hong Kong/483/97 (H5N1) (11689-V08H, Sino Biological Inc.), A/Shanghai/1/2013 (H7N9) (40104-V08H, Sino Biological Inc.), or A/Hong Kong/1073/99 (H9N2) (11229-V08H, Sino Biological Inc.). Plates were blocked as above and incubated overnight at 4°C with diluted serum samples from individual mice. Next, plates were incubated with either alkaline phosphatase conjugated anti-mouse IgG (A1418, Sigma-Aldrich, Germany), biotinylated anti-IgG1^a^ (553599, BD Pharmingen), or biotinylated anti-IgG2a^a^ (553502, BD Pharmingen), and developed as above. For some ELISAs (Figure 5), the detection was performed with horseradish peroxidase-conjugated anti-mouse IgG (115-035-071, Jackson ImmunoResearch) and the 1-Step UltraTMB substrate (Thermo Fisher Scientific). Plates were then read at 450 nm with the VersaMax reader (MDS Analytical Technologies).

### Virus

The influenza virus A/Puerto Rico/8/34 (mt.Sinai sub-strain) (H1N1) (PR8) was a kind gift from Dr. Anna Germundsson at the National Veterinary Institute, Norway. The influenza virus A/turkey/Italy/3889/1999 (H7N1) was a kind gift from Professor Rebecca Cox at the University of Bergen, Norway. Prior to use, the H7N1 virus was mouse adapted by several passages in BALB/c mice.

### Mouse Immunization and Challenge

Plasmids were purified by Endofree Qiagen kit (Qiagen, the Netherlands) and dissolved in NaCl.

BALB/c mice were anesthetized [0.1 mg/10 g body weight with cocktail of: Zoletil Forte (250 mg/ml) (Virbac France), Rompun (20 mg/ml) (Bayer Animal Health GmbH), and Fentanyl (50 μg/ml) (Actavis, Germany)] by intraperitoneal (i.p.) injection and vaccinated by intramuscular (i.m.) injection with a total of 200 μg of plasmid DNA into the quadriceps muscles, immediately followed by electroporation (EP) (Elgen, Inovio, USA) of the injection site. For viral challenge, BALB/c mice were anesthetized as above and given intranasal (i.n.) inoculations of virus in 10 μl per nostril. Mice were monitored for weight loss relative to the day of challenge (day 0), with an endpoint of a 20% weight reduction as required by NARA.

F1 of BALB/c and knock-in CR9114 gH mice (C57BL/6 background) were anesthetized by isoflurane and vaccinated by i.m. injection with a total of 200 μg DNA into the quadriceps muscles immediately followed by EP (TriGrid Delivery system, Ichor Medical Systems). For viral challenge, the mice were anesthetized as above and given intratracheal (i.t.) inoculations of 2.5 × LD50 in 50 μl. Mice were monitored for weight loss relative to the day of challenge (day 0), with an endpoint of 25% weight loss.

### T Cell Depletion

BALB/c mice were vaccinated as described above. Starting at day 12 after the second vaccination and until termination, mice that received αMHCII-H1stem were injected every other day i.p. with 200 μg of purified anti-CD4 (GK1.5, ATCC) and anti-CD8 (TIB105, ATCC), or control mAbs (SRF8-B6 and Y13-238). On day 14, mice were challenged with PR8 and monitored for weight loss. In order to assess the degree of depletion, spleens were harvested at termination and stained for FACS analysis with the following mAbs: Pacific Blue-conjugated rat anti-mouse CD8a (558106, BD Pharmingen, CA, UA), PE-conjugated rat anti-mouse CD3ε (100308, BioLegend, CA, US), and PerCP/Cy5.5-conjugated rat anti-mouse CD4 (100434, BioLegend). In addition, the following isotype-matched control mAbs were used: Pacific Blue-conjugated rat IgG2aκ (558109, BD Pharmingen) and PerCP/Cy5.5-conjugated rat IgG2bκ (400632, BioLegend).

### Hybridoma Generation and Transfer to Mice

Splenocytes collected from vaccinated mice were fused with mouse plasmacytoma cells (OURI) using polyethylene glycol (Roche, Mannheim, Germany) as previously described (27). After fusion, cells were cultured in 96-well plates with RPMI 1640 containing 10% fetal bovine serum (Gibco, Life Technologies, Carlsbad, CA, USA) and supplemented with hypoxanthine-aminopterin-thymidine (HAT) (Sigma-Aldrich, St. Louis, MO, USA). Limiting dilutions were performed on positive colonies detected in ELISA from day 14 or 18 after fusion. The ELISAs were set up with coats of HA protein from influenza PR8 (11684-V08H, Sino Biological Inc., USA) or A/Hong Kong/483/97 (H5N1) (11689-V08H, Sino Biological Inc.), or ovalbumin (OVA) (A5503, Sigma), or by coating with Phox-BSA and recombinant proteins expressing Phox-specific scFv linked to the HA stem. Detection was performed with either alkaline phosphatase-conjugated anti-mouse IgG (A1418 Sigma-Aldrich) or biotinylated anti-IgM (553515, BD Pharmingen) and streptavidin alkaline phosphatase (1:3000, GE Healthcare). The positive colonies were positive for IgM. Plates were developed with phosphatase substrate (P4744-10G, Sigma-Aldrich) and read at 405 nm with a Tecan reader using the Magellan v5.03 program.

Cells of 3-H7 (see Supplementary Figure S3A) and isotype-matched 167.7 (negative control) were expanded in cell culture, and the produced mAb was affinity purified on a column with mAb 187.1 (anti-κ). Mice were injected i.p. with 200 μg of either 3-H7 or 167.7 in 400 μl of NaCl. The next day, mice were challenged with a 5 × LD50 dose of influenza PR8 virus and monitored for weight loss relative to the day of challenge (day 0).

### Statistical Analyses

Statistical analyses of antibody responses in sera were performed using two-way ANOVA and Bonferroni's multiple comparison test. All other analyses were performed using the non-parametric Mann–Whitney test (GraphPad Software Inc.). The alpha level was set to 0.05 for all analyses.

## Results

### Construction and Characterization of Vaccine Molecules

**Figure 1:**
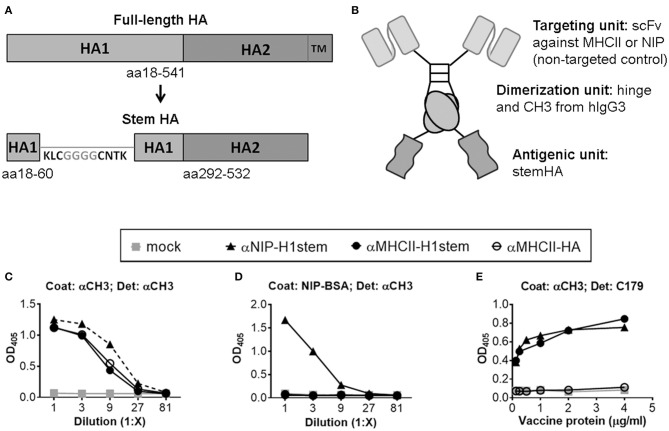
Characterization of vaccine molecules. **(A)** Schematic of the linear structure of the full-length HA protein (top), and the vaccine-inserted HA stem domain (bottom). Inserted amino acids are shown in gray, while amino acids present in the native HA sequence are in black. **(B)** Schematic structure of a dimeric vaccine protein. **(C,D)** Supernatants of transfected 293E cells were examined by ELISAs using either a mAb against C_H_3 **(C)** or NIP-BSA **(D)** as coat. Proteins were detected with a biotinylated mAb against C_H_3. **(E)** Purified vaccine proteins were assayed in an ELISA coated with a mAb against C_H_3, and detected with mAb against the stem region of HA (C179).

The stem regions of HA from influenza virus A/PuertoRico/8/1934 (H1N1) (PR8) (aa 18–60 and 292–532, numbering according to H0) were joined by a linker that separated a conserved disulfide bridge in the stem region by four glycines (KLCGGGGCNTK), as previously described (11) (Figure 1A). Next, the HA stem was inserted into a vaccine construct that linked the HA stem, via a dimerization unit consisting of the hinge and C_H_3 exons of human IgG3, to a targeting unit consisting of a single-chain variable fragment (scFv) specific for mouse MHC class II molecules (I-E^d^) (Figure 1B) (19, 20). As non-targeted control, a similar vaccine was prepared, but where the targeting unit was replaced with a scFv specific for the hapten NIP. The vaccines were denoted αMHCII-H1stem and αNIP-H1stem, respectively.

The constructs were inserted into pUMVC vectors (26), and the vaccine plasmids were transiently transfected into 293E cells for confirmation of efficient secretion of the vaccine proteins (Figures 1C,D). For further assessment of antigenic integrity, we tested whether the stem reactive mAb C179 (5) could recognize the inserted antigen. Previously, C179 has been described to neutralize influenza viruses by blocking membrane fusions (5) and to bind HA stem residues from both influenza groups 1 and 2 (28). Importantly, the mAb C179 bound the vaccine constructs expressing stem HA as antigen (Figure 1E), thus demonstrating that the antigen was recognized by a biologically significant mAb. In contrast, the mAb C179 did not bind a control vaccine expressing the full extracellular domain of HA (aa 18–541), αMHCII-HA, indicating that the HA stem domain is not easily accessible in this format.

### Targeting of HA Stem to MHC Class II Molecules Induces Protective Antibody Responses

**Figure 2:**
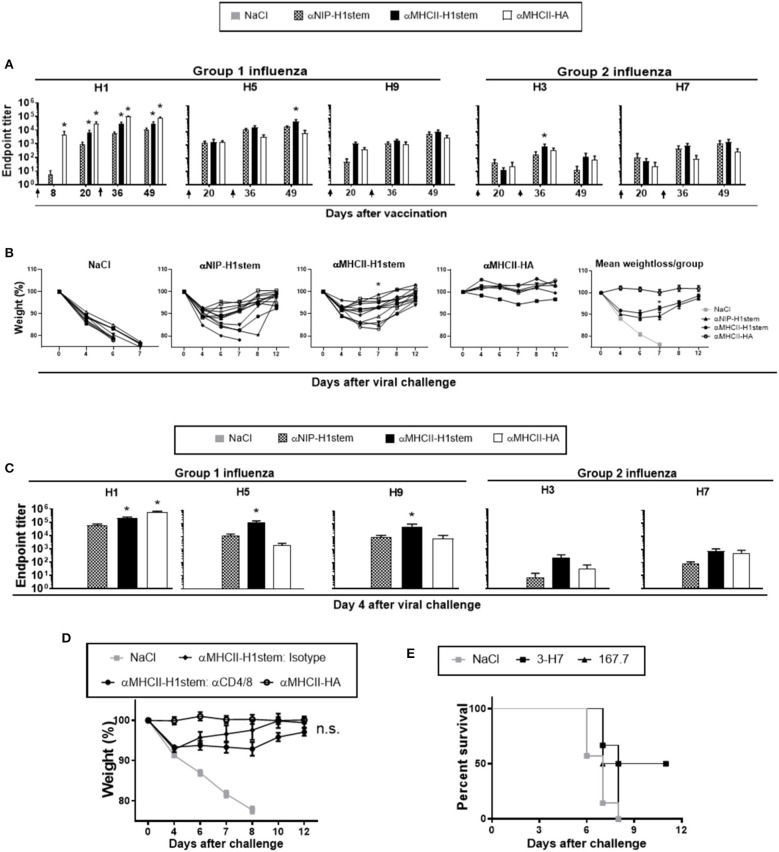
Induction of cross-reactive and protective antibodies against influenza. **(A–C)** BALB/c mice (*n* = 11–12 mice/group, except *n* = 6 for the αMHCII-HA group) were DNA-immunized twice on days 0 and 21, as indicated by arrows. **(A)** Sera were analyzed for development of IgG responses against recombinant HA from PR8, A/Hong Kong/1/1968 (H3N2), A/Hong Kong/483/97 (H5N1), A/Shanghai/1/2013 (H7N9), and A/Hong Kong/1073/99 (H9N2). A vaccine encoding full-length HA, αMHCII-HA, was included as positive control. Values given are mean ± SEM. *p < 0.05 as compared to αNIP-H1stem (two-way ANOVA and Bonferroni post-test). **(B)** 4 weeks after the final vaccination, mice were challenged with a 5 × LD50 dose of influenza PR8 and monitored for weight loss. *p < 0.05 for αMHCII-H1stem as compared to αNIP-H1stem (Mann–Whitney test). **(C)** Sera were harvested on day 4 after the influenza challenge and assayed for specific IgG responses against HA from PR8, A/Hong Kong/1/1968 (H3N2), A/Hong Kong/483/97 (H5N1), A/Shanghai/1/2013 (H7N9), and A/Hong Kong/1073/99 (H9N2) in ELISA. Values given are mean ± SEM. *p < 0.05 as compared to αNIP-H1stem (two-way ANOVA and Bonferroni post-test). **(D)** BALB/c mice were immunized twice (day 0 and 21) and then injected every other day with depleting mAbs against CD4^+^- and CD8^+^-T cells starting from day 48 (*n* = 7 for αMHCII-HA, *n* = 8 for isotype treated, *n* = 11 for CD4/8 depleted, and *n* = 10 for NaCl). At day 50, mice were challenged with a 5 × LD50 dose of influenza PR8, and monitored for weight loss. **(E)** Hybridomas were generated after vaccination with αMHCII-H1stem (S5). Survival of BALB/c mice that were treated i.v. with monoclonal IgM 3-H7 specific for the HA stem or a matched isotype control (167.7), and challenged 24 h later with a 5 × LD50 dose of influenza PR8.

BALB/c mice were injected twice intramuscularly (i.m.) with vaccine plasmids encoding stem HA, or a positive control encoding full-length HA from influenza PR8 (αMHCII-HA) (20). Immediately after DNA injection, EP was applied to the injection site in order to increase DNA uptake (29). A single immunization with αMHCII-H1stem significantly increased serum levels of antibodies specific for HA from PR8, as compared to αNIP-H1stem (Figure 2A, left panel). The responses were further boosted by a second vaccine delivery, and the antibody titers in the group receiving αMHCII-H1stem remained significantly higher than after vaccination with αNIP-H1stem. The positive control vaccine, αMHCII-HA, has previously been demonstrated to confer antibody-mediated protection as soon as 14 days after a single DNA vaccination (20). As expected, vaccination with this vaccine greatly enhanced antibody responses after a single DNA delivery, with a further elevation after the boost.

In order to test the cross-reactive potentials of the vaccine-induced antibodies, sera were assayed against recombinant HA proteins from H3, H5, H7, and H9 (Figure 2A) influenza viruses in ELISA. While a single immunization could induce detectable antibodies in sera, a boost further increased the antibody levels. In correspondence with results from the H1 ELISA, vaccination with αMHCII-H1stem was more efficient than αNIP-H1stem at inducing cross-reactive antibodies, but the difference was only significant for HA from H1, H3, and H5 influenza viruses. As would be expected, vaccination with the control αMHCII-HA could only raise very low levels of cross-reactive antibodies.

Following two immunizations with a 3 week interval, mice were challenged 4 weeks later with influenza PR8 virus. Disease progression was monitored by weight changes relative to the day of infection (day 0) (Figure 2B). While an initial weight loss was observed in all mice receiving vaccines encoding the HA stem domain, a significant improvement in disease recovery was observed on day 7 in mice vaccinated with αMHCII-H1stem as compared to αNIP-H1stem (Figure 2B and Supplementary Figure S1A). When comparing αMHCII-H1stem to αMHCII-HA, however, it was clear that the mechanism of protection was different. While αMHCII-HA can induce strain-specific sterilizing immunity against influenza (20), the initial weight loss found after vaccination with αMHCII-H1stem indicated that the induced antibodies could not completely block infection.

To get a more clear picture of the protective mechanisms triggered by the different vaccines, sera were collected 4 days after viral challenge and analyzed for antibody responses against recombinant HA proteins from influenza H1, H3, H5, H7, and H9 (Figure 2C). Results demonstrated that the virus challenge had particularly boosted antibody responses in mice vaccinated with αMHCII-H1stem, as compared to αNIP-H1stem. While the antibody levels reactive against H1, H5, and H9 influenza viruses were significantly increased in mice vaccinated with αMHCII-H1stem, the viral challenge would only enhance antibodies specific for H1 influenza in mice vaccinated with αMHCII-HA. Responses against HA from H3 and H7 influenza viruses remained low in all vaccine groups (Figure 2C). This is interesting because H1, H5, and H9 phylogenetically belong to group 1 influenza, whereas H3 and H7 belong to group 2. The two groups are characterized by structural differences in the stem domain (30). Thus, the antibodies induced by MHCII-targeted delivery of the H1-derived stem domain seem to be restricted to group 1 influenza. Consistent with this, vaccination with αMHCII-H1stem failed to confer protection against a lethal challenge with influenza H7N1 (Supplementary Figure S1B).

In order to examine if the increased antibody responses observed after vaccination and viral challenge with αMHCII-H1stem could indeed contribute to the observed protection, experiments were set up to examine antibody functionality in the absence of T cell responses. In a first experiment, mice were vaccinated as above and then treated with T cell-depleting antibodies every second day from 2 days prior to a viral challenge with influenza PR8 (Figure 2D and Supplementary Figure S2). No significant differences were observed between αMHCII-H1stem-vaccinated mice injected with either depleting antibodies against CD4^+^ and CD8^+^ T cells or isotype-matched control antibodies, indicating a contribution of the vaccine induced antibodies to protection.

To more directly test the influence of antibodies, B cell hybridomas were obtained from mice vaccinated twice with αMHCII-H1stem. The cell lines were screened for the presence of antibodies against HA from influenza viruses H1, H5, and H7, and also against the HA stem domain and ovalbumin (Supplementary Figure S3A). A candidate cell line was then selected for upscaling and antibody production. The purified mAbs were injected into naïve mice that were challenged the next day with influenza PR8 virus. Importantly, the stem-specific mAbs induced by αMHCII-H1stem could confer protection in about half of the mice, as compared to isotype-matched control antibodies (Figure 2E and Supplementary Figure S3B). Thus, while we cannot rule out a contribution from T cells to protection after vaccination with αMHCII-H1stem, the vaccine-induced antibodies clearly contributed to protection against influenza.

### Targeting of H1stem to Chemokine Receptors or MHCII Induces Similar Protection

We have previously demonstrated that selective targeting of antigens to different receptors on APC can polarize immune responses to different types (23). As an example, targeting of antigen to MHCII molecules induced an antibody response that was dominated by IgG1. By contrast, targeting of antigen to chemokine receptors 1, 3, and 5 (CCR1/3/5) with the chemokine MIP1α induced an antibody response more polarized to IgG2a. Since IgG2a has a higher affinity for Fcγ receptors than IgG1 and, unlike IgG1, can fix complement, IgG2a antibodies can more efficiently mediate effector functions such as antibody-dependent cellular cytotoxicity (ADCC) and complement-dependent cytotoxicity (CDC). As such, the antibody response could potentially be broader in nature since more antibody specificities could contribute to protection (31). To test if targeting of the stem domain to CCR1/3/5 could lead to more efficient protection against influenza, we constructed a vaccine where the MHCII-specific targeting unit was replaced with MIP1α (MIP1α-H1stem).

**Figure 3:**
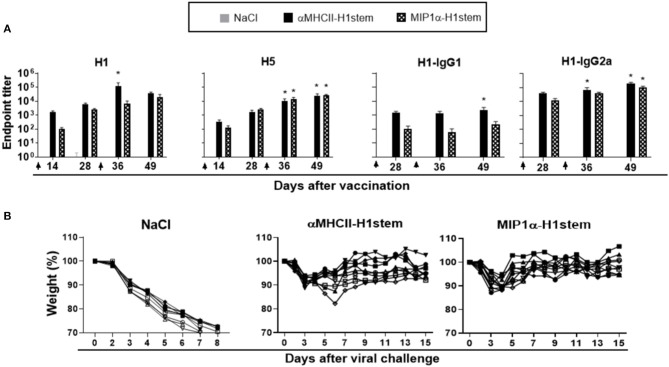
Antibodies and protective efficacy after vaccination with αMHCII-H1stem and MIP1α-H1stem. BALB/c mice (*n* = 10 mice/group) were DNA-immunized twice on days 0 and 29, as indicated by arrows. **(A)** Sera were analyzed for development of total IgG responses against recombinant HA from PR8 and A/Hong Kong/483/97 (H5N1) and IgG1/IgG2a against HA from PR8. Values given are mean ± SEM. *p < 0.05 as compared to NaCl (two-way ANOVA and Bonferroni post-test). **(B)** 4 weeks after the final vaccination, mice were challenged with a 5 × LD50 dose of influenza PR8 and monitored for weight loss.

Mice were immunized with DNA twice with a 4 week interval, and antibody responses were assayed againt influenza viruses H1, H5, H3, and H7 in ELISA (Figure 3A and Supplementary Figure S4A). As expected (23), αMHCII-H1stem induced higher antibody responses than MIP1α-H1stem against H1 influenza virus, but the vaccines induced similar responses to the H5 influenza virus. The IgG response detected after vaccination with αMHCII-H1stem was dominated by IgG1 (Figure 3A). Neither vaccine induced significant antibody responses against HA from H3 and H7 influenza viruses (Supplementary Figure S4A). Despite the differences in antibody responses observed for these two vaccines, both αMHCII-H1stem and MIP1α-H1stem conferred protection against influenza to a similar extent (Figure 3B).

### Development of Vaccines Against Both Group 1 and 2 Influenza Viruses

As described above, the immune responses and protection induced by vaccinations with either αMHCII-H1stem or MIP1α-H1stem were limited to efficacy against subtypes of group 1 influenza viruses. In order to develop a vaccine capable also of mediating protection against group 2 influenza viruses, fragments encoding the stem regions of HA from influenza A/Shanghai/2/2013 (H7N9) (aa 19–61 and 289–535) were inserted into the MHCII-targeted vaccine format (αMHCII-H7stem). Assessments of supernatants from 293E cells transiently transfected with the new vaccine construct demonstrated a secretion level similar to that of αMHCII-H1stem (Supplementary Figure S4B).

**Figure 4:**
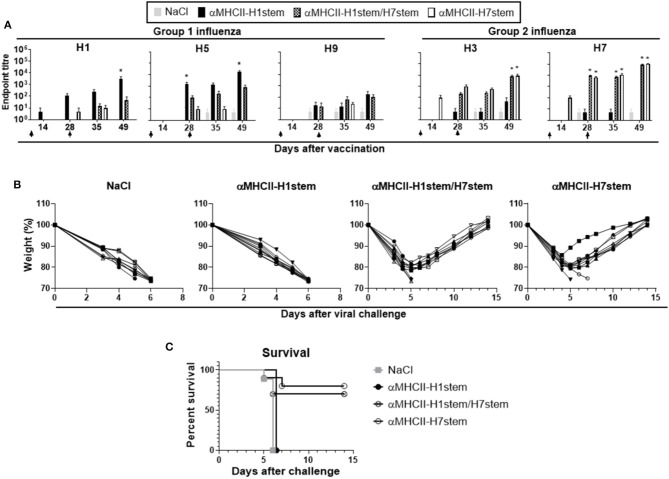
Vaccination and protection against challenge with H7N1 influenza. **(A)** BALB/c mice (*n* = 10 mice/group) were immunized on days 0 and 28, as indicated by arrows, and sera analyzed for development of IgG responses against recombinant HA from PR8 (H1N1), A/Hong Kong/1/1968 (H3N2), A/Hong Kong/483/97 (H5N1), A/Shanghai/1/2013 (H7N9), and A/Hong Kong/1073/99 (H9N2). Values given are mean ± SEM. *p < 0.05 as compared to NaCl (two-way ANOVA and Bonferroni post-test). **(B)** At day 49 after vaccination, mice were challenged with 5 × LD50 of influenza A/turkey/Italy/3889/1999 (H7N1) and monitored for weight **(B)** and survival **(C)**.

Mice were immunized with a total of 200 μg of DNA plasmids encoding either αMHCII-H7stem, αMHCII-H1stem, or both (αMHCII-H1stem/H7stem), and antibody responses were assayed against recombinant HA proteins from H1, H3, H5, H7, and H9 influenza viruses (Figure 4A). As expected, αMHCII-H1stem induced significant antibody responses against HA from H1, H5, and H9 of group 1 influenza viruses, whereas αMHCII-H7stem significantly induced antibody responses against H3 and H7 of group 2 influenza viruses. Interestingly, whereas the mix vaccine αMHCII-H1stem/H7stem was a poor inducer of antibody responses against group 1 influenza viruses, it raised antibody levels against HA from H3 and H7 influenza viruses to levels comparable to those of mice immunized with αMHCII-H7stem. The reason for this was not further evaluated but is likely due to inherent differences in immunogenicity between the stem region of H1 and H7 influenza viruses.

Mice were vaccinated twice with a 4 week interval and challenged with a lethal dose of influenza A/turkey/Italy/3889/1999 (H7N1). Weight was assessed as a marker of disease (Figure 4B). As expected, vaccination with αMHCII-H1stem could not protect against the challenge with H7N1 influenza virus, but vaccination with αMHCII-H7stem conferred protection in 8/10 mice against the lethal challenge. In line with the similar antibody responses induced against HA from group 2 influenza viruses after vaccination with αMHCII-H7stem and αMHCII-H1stem/H7stem, 7/10 mice were also protected after vaccination with αMHCII-H1stem/H7stem (Figure 4C).

### Vaccination and Protection in Mice Expressing Human HA Stem-Reactive BCR

**Figure 5:**
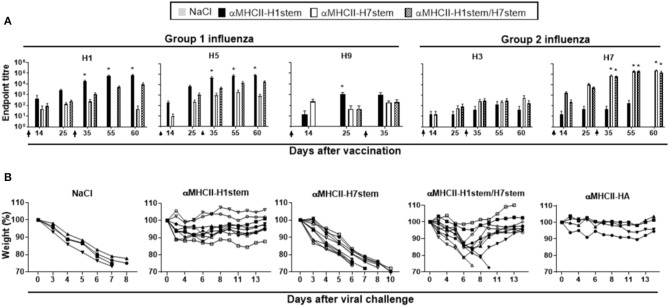
Protection against influenza H1N1 in F1 mice of BALB/c and CR9114 gH knock-in. **(A)** F1 of BALB/c and knock-in 9,114 gH mice (*n* = 10 mice/group, *n* = 4 mice/group for NaCl and αMHCII-HA) were immunized twice on days 0 and 28, as indicated by arrows, and sera analyzed for development of IgG responses against recombinant HA from PR8 (H1N1), A/Hong Kong/1/1968 (H3N2), A/Hong Kong/483/97 (H5N1), A/Shanghai/1/2013 (H7N9), and A/Hong Kong/1073/99 (H9N2). Values given are mean ± SEM. *p < 0.05 as compared to NaCl (two-way ANOVA and Bonferroni post-test). **(B)** At day 59 after vaccination, mice were challenged with 2.5 × LD50 of influenza A/PR/8/1934 (H1N1) and monitored for weight.

About 80% of the stem-reactive antibodies that have been observed in humans use a particular H-chain variable region called V_H_1-69 (32) and one of the mAbs that uses this gene has been demonstrated to bind with high affinity to most, if not all, influenza A strains (CR9114) (25). Moreover, this antibody binds the HA stem solely through H-chain contacts (25). Therefore, we set up an experiment in CR9114 gH mice, in which the inferred unmutated version of the CR9114 VDJ_H_ and an upstream Vh promoter were knocked into the J_H_ locus of C57BL/6 mice. Although B cells with this H-chain have very low affinity for HA, they are predicted to have the potential to produce broadly neutralizing stem antibodies with appropriate mutation. To allow targeting by the MHCII-targeting immunogen, we generated F1 hybrids of CR9114 gH homozygous mice with BALB/c. The mice were vaccinated as above with αMHCII-H1stem, αMHCII-H7stem, or both (αMHCII-H1stem/H7stem). An analysis of antibody responses in sera against HA from H1, H3, H5, H7, and H9 influenza viruses demonstrated that while αMHCII-H1stem induced antibodies against the group 1 influenza subtypes H1, H5, and H9, αMHCII-H7stem induced antibodies against H3 (albeit weak) and H7 (Figure 5A). Vaccination with αMHCII-H1stem/H7stem significantly raised antibody responses against HA from influenza H3 and H7 (Figure 5A), as was also demonstrated above in BALB/c mice (Figure 4A).

At week 4 after vaccination, mice were challenged with influenza PR8 virus and monitored for weight loss (Figure 5B). Importantly, all mice that were vaccinated with αMHCII-H1stem were protected, and also 8/10 of the mice were vaccinated with αMHCII-H1stem/H7stem. By contrast, mice vaccinated with αMHCII-H7stem succumbed to the infection.

### Repeated Delivery of Stem-Based Vaccine or Booster Delivery With a Vaccine Mixture

For the above experiments, we have used the stem-encoding DNA vaccines for both prime and boost vaccinations. Previously, we have demonstrated that vaccination with a mixture of full-length HAs from six different subtypes of influenza (H5, H6, H8, H9, H11, and H13) could induce broadly reactive protective antibodies against HA from an influenza subtype that was not included in the vaccine mixture (33). Thus, we here wanted to test how a booster vaccination with this vaccine mixture (αMHCII-MIX), or a non-targeted control vaccine mixture (αNIP-MIX), would compare to two successive vaccination with αMHCII-H1stem/H7stem.

**Figure 6:**
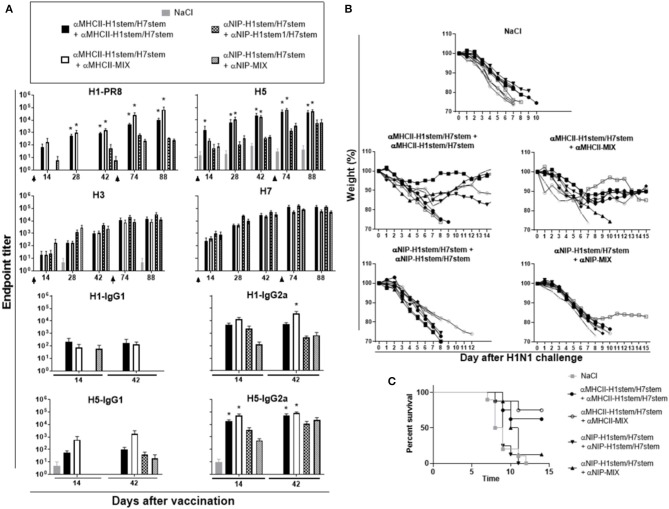
Protection after boost with HA stem domain of MHCII-targeted vaccine mixture. **(A)** F1 of BALB/c and knock-in 9,114 gH mice (*n* = 8 mice/group) were immunized twice, as indicated by arrows, and sera analyzed for development of IgG responses against recombinant HA from PR8 (H1N1), A/Hong Kong/1/1968 (H3N2), A/Hong Kong/483/97 (H5N1), A/Shanghai/1/2013 (H7N9), and A/Hong Kong/1073/99 (H9N2). Values given are mean ± SEM. *p < 0.05 as compared to the corresponding non-targeted control, αNIP-H1stem/H7stem+αNIP-H1stem/H7stem, or αNIP-H1stem/H7stem+αNIP-MIX (two-way ANOVA and Bonferroni post test). **(B,C)** 10 weeks after the second vaccination, mice were challenged with 2.5 × LD50 of influenza A/PR/8/1934 (H1N1), and monitored for weight **(B)** and survival **(C)**.

CR9114 knock-in mice were immunized either twice with αMHCII-H1stem/H7stem or αNIP-H1stem/H7stem or vaccinated once with the stem-encoding DNA vaccines followed by a boost with either αMHCII-MIX or αNIP-MIX. Interestingly, the antibody responses elicited by two deliveries of αMHCII-H1stem/H7stem were similar to the levels induced after a boost with αMHCII-MIX, and the only significant difference in antibody induction was the increased levels of IgG2a that were observed against HA from influenza H1 (Figure 6A). While MHCII targeting of the vaccines was necessary for raising antibody responses against H1 and H5, the non-targeted control vaccines (αNIP-H1stem/H7stem and αNIP-MIX) were sufficient for raising antibodies against HA from influenza H3 and H7 subtypes.

At day 70 after the boost, mice were challenged with influenza PR8 virus and monitored for weight (Figure 6B) and survival (Figure 6C). While there was a significant difference between MHCII-targeted vaccination and the non-targeted control vaccines (αNIP-H1stem/H7stem and αNIP-MIX), there were no significant differences between two deliveries with αMHCII-H1stem/H7stem or the booster delivery with αMHCII-MIX.

## Discussion

Given the potential of stem-directed mAbs to mediate cross-protection against a multitude of influenza strains, such antibodies are currently popular for clinical applications (34). We have here demonstrated that a DNA vaccine can be used for induction of protective and broadly reactive antibodies against the HA stem domain. This is important because the DNA format allows for large-scale production and mass vaccinations. The HA stem domain is a weak antigen and DNA vaccines have typically been hampered by reduced immunogenicity. Here, we increased vaccine immunogenicity and efficacy by targeting the HA stem domain to MHCII molecules on APC after translation of the injected DNA to vaccine proteins. Importantly, we have also developed a human version of the MHCII-specific targeting unit, capable of pan-specifically binding human leukocyte antigen class II (HLAII) molecules (21). Thus, the strategy is feasible for prophylactic use in the human population.

We here demonstrated that vaccination with αMHCII-H1stem significantly increased the induced levels of cross-reactive antibodies, as compared to the non-targeted control, αNIP-H1stem. These two constructs are similar in size, structure, and antigenic content. The observed difference in immunogenicity could thus be explained by the increased efficacy typically associated with APC targeting (35–38). However, while we also observed a significant protective efficacy of APC targeting as compared to vaccination with the non-targeted control (Figure 2), the difference was more subtle than the observed antibody levels would indicate. It is possible that the xenogeneic sequences in the dimerization unit has augmented immunity and that more T cells have been induced in the absence of MHCII targeting (39).

Previously, we demonstrated that a single DNA vaccination with MHCII-targeted HA could confer sterilizing immunity against a homologous strain of influenza virus (20). In the present study, we observed that mice immunized with αMHCII-H1stem experienced a more pronounced weight loss after challenge with influenza viruses, as compared to mice immunized with αMHCII-HA. The weight loss indicated that the mechanism of protection was different here, but the experiment in which T cells were depleted prior to viral challenge demonstrated that the vaccine-induced antibodies certainly contribute to the observed protection. Others have demonstrated that stem-specific antibodies can block viral replication by locking the HA in its pre-fusion conformation and as such hamper the fusion between the virus and host membranes, or by inhibiting viral exit (7–9, 25). Furthermore, FcγR interactions could mediate viral clearance by ADCC (40), CDC (41), or antibody-dependent cell phagocytosis (ADCP) (42). The protective mechanism of action after vaccinations with MHCII-targeted HA stem domains remains unclear because targeting toward CCR1/3/5 with its increased IgG2a polarization of the vaccine-induced antibodies did not improve protective efficacy (Figure 3).

The first stem-specific antibody was isolated from BALB/c mice immunized with H2N2 influenza virus in 1993 (5). Since then, many such antibodies have been discovered. Typically, the stem-specific antibodies can cross-react with other influenza subtypes belonging to the same influenza group (group 1; H1, H2, H5, H6, H11, H13, H16, H17, and H18, and group 2: H3, H4, H14, H7, H10, and H15), but some rare examples of antibodies able to broadly bind subtypes from both influenza groups 1 and 2 have also been described (25, 43). Here, we observed that vaccinations with αMHCII-H1stem and αMHCII-H7stem induced antibodies against HA from the corresponding group of influenza and that protective efficacy was restricted to the same group. Thus, we vaccinated mice with a mixture of αMHCII-H1stem and αMHCII-H7stem, and demonstrated that this mixture significantly increased the breadth of protection against influenza viruses from both group 1 and group 2. That said, the efficacy of the protection was consistently better after vaccination with either αMHCII-H1 stem or αMHCII-H7stem. The antigenic content of each vaccine was lower in the mixture as compared to separate vaccinations with each of the vaccines, which is a likely explanation for this difference.

The APC-targeted vaccine format is likely to display the HA stem domain in a monomeric form (20) and where the HA stem integrity is only maintained by the intrinsically conserved disulfide bridge located to the membrane-distal end. The structural integrity of the stem domain is therefore uncertain, and it is not known if the induced antibodies bind conformational of linear epitopes. Presumably, the monomeric stem domain protrudes from the dimerization unit and, as such, makes antigenic sites accessible for recognition by B cell receptors (BCRs). Furthermore, the APC-targeted vaccine format will bivalently display the HA stem and, as such, potentially promote cross-linking of BCR that are specific for antigenic determinants located to the stem domain. Hypothetically, αMHCII-HAstem proteins could form an APC-B cell synapse by bridging BCR with MHCII molecules expressed on APC and, as such, efficiently increase activation of both the B cell and CD4^+^ helper T cells (39, 44).

CR9114 gH mice made anti-stem responses comparable to wild-type mice despite their high frequency of potential B cell precursors. As its B cells mainly have no or very low affinity for HA stem, we speculate that immunogen designs that target this class of germline BCRs with higher affinity might be needed to improve the response.

The HA stem domain remains a highly interesting target for development of more universal influenza vaccines. The present study adds knowledge important for development of a universal influenza vaccine by demonstrating that the HA stem can be delivered in the form of DNA. This is important because production of recombinant HA stem proteins is tedious and cannot present the quanta required for large-scale prophylactic vaccinations. Further, the DNA format allows for cheaper vaccines that can be distributed independently of a cold chain.

## Data Availability Statement

All datasets generated for this study are included in the article/Supplementary Material.

## Ethics Statement

The animal study was reviewed and approved by Norwegian Animal Research Authority (NARA), and the Institutional Animal Care and Use Committee at The Scripps Research Institute.

## Author Contributions

GG, BB, and DN conceived and designed experiments. GG, MB-H, JA, TB, and JT performed experiments. GG, MB-H, and BB analyzed the experiments and wrote the paper. All authors commented and edited on the paper.

### Conflict of Interest

BB is an inventor on patent applications filed on the vaccine molecules by the TTO offices of the University of Oslo and Oslo University Hospital, according to institutional rules. BB is head of the Scientific panel of Vaccibody AS, and holds shares in the company. The remaining authors declare that the research was conducted in the absence of any commercial or financial relationships that could be construed as a potential conflict of interest.
