# Supplementary material for: Induction of Cross-Reactive and Protective Antibody Responses After DNA Vaccination With MHCII-Targeted Stem Domain From Influenza Hemagglutinin
Source: Front Immunol. 2020 Mar 25;11:431. doi: 10.3389/fimmu.2020.00431 (PMC7112135; doi:10.3389/fimmu.2020.00431)
Supplement: Supplementary file 1 [file Data_Sheet_1.PDF]

Supplemental figures

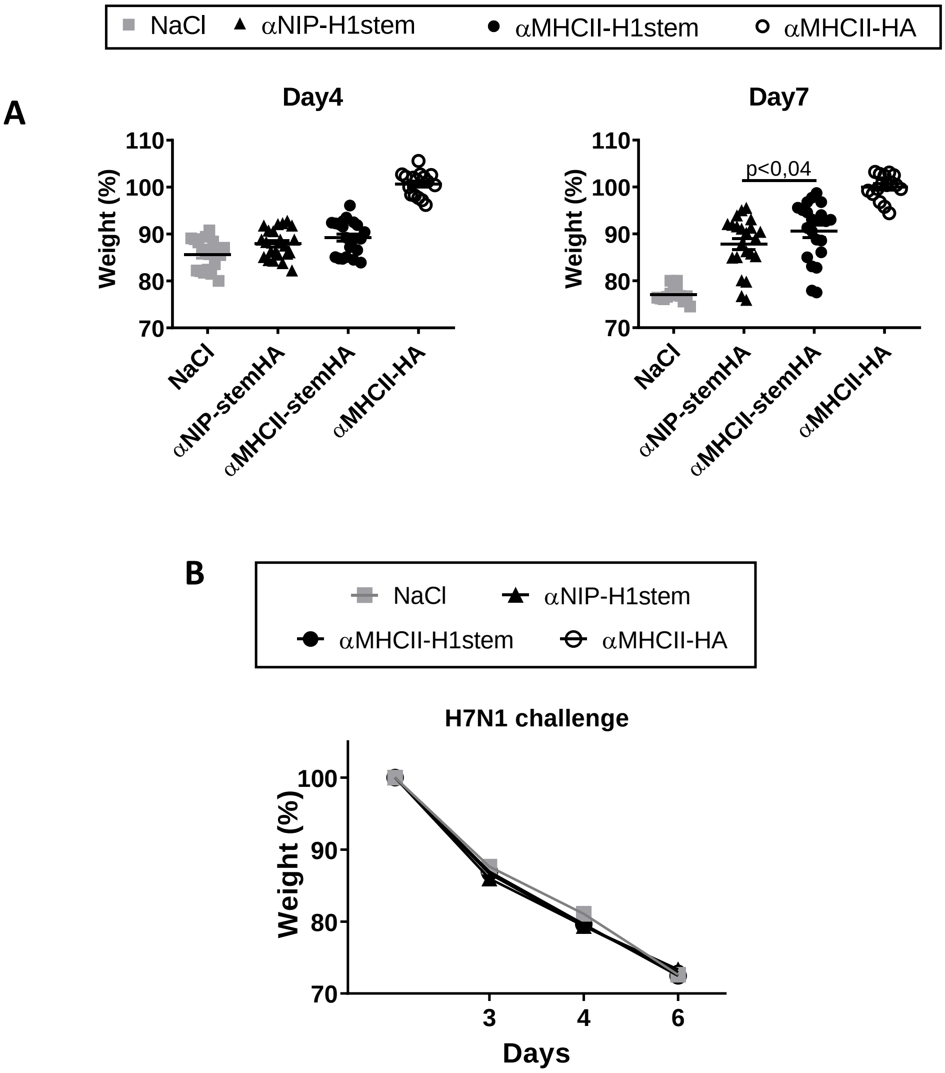

S1. Protection against viral challenge with influenza PR8 and H7N1. (A) BALB/c mice (n=16-22 mice/group) were vaccinated twice with a 3 week interval, and then challenged 4 weeks after the final vaccination with a 5xLD50 dose of influenza PR8. Mice were monitored for weightloss, with day 4 and day 7 after challenge depicted. *indicates $p<0.05$ as compared to αNIP-H1stem (Mann-Whitney test). (B) Mice (n=6/group) were immunized on days 0 and 21, and then challenged with 5xLD50 of influenza H7N1 on day 50.

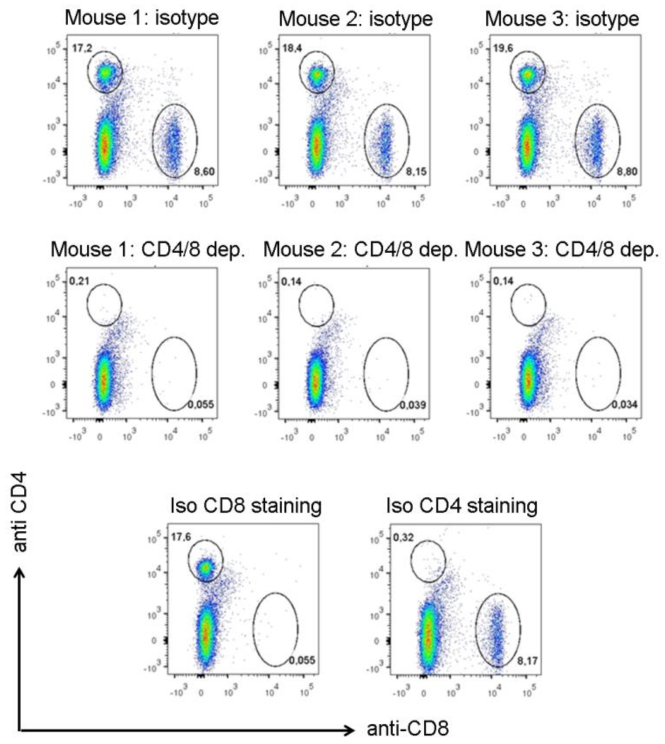

S2. Efficacy of T cell depletion in Figure 2D. Mice were immunized twice on days 0 and 21, and treated every other day from day 48 with depleting antibodies against CD4 (GK1.5) and CD8 (TIB105), or isotype controls. Splenocytes were harvested at the termination of the experiment (day 62) to assess the degree of depletion in mice receiving either isotype control mAbs (upper row) or depleting mAbs (middle row). The samples are representative within groups. As an additional control, splenocytes from mice treated with isotype matched antibodies were stained with antibodies isotype matched to the mAbs used for staining of $CD8^+$ and $CD4^+$ T cells, respectively (lower row).

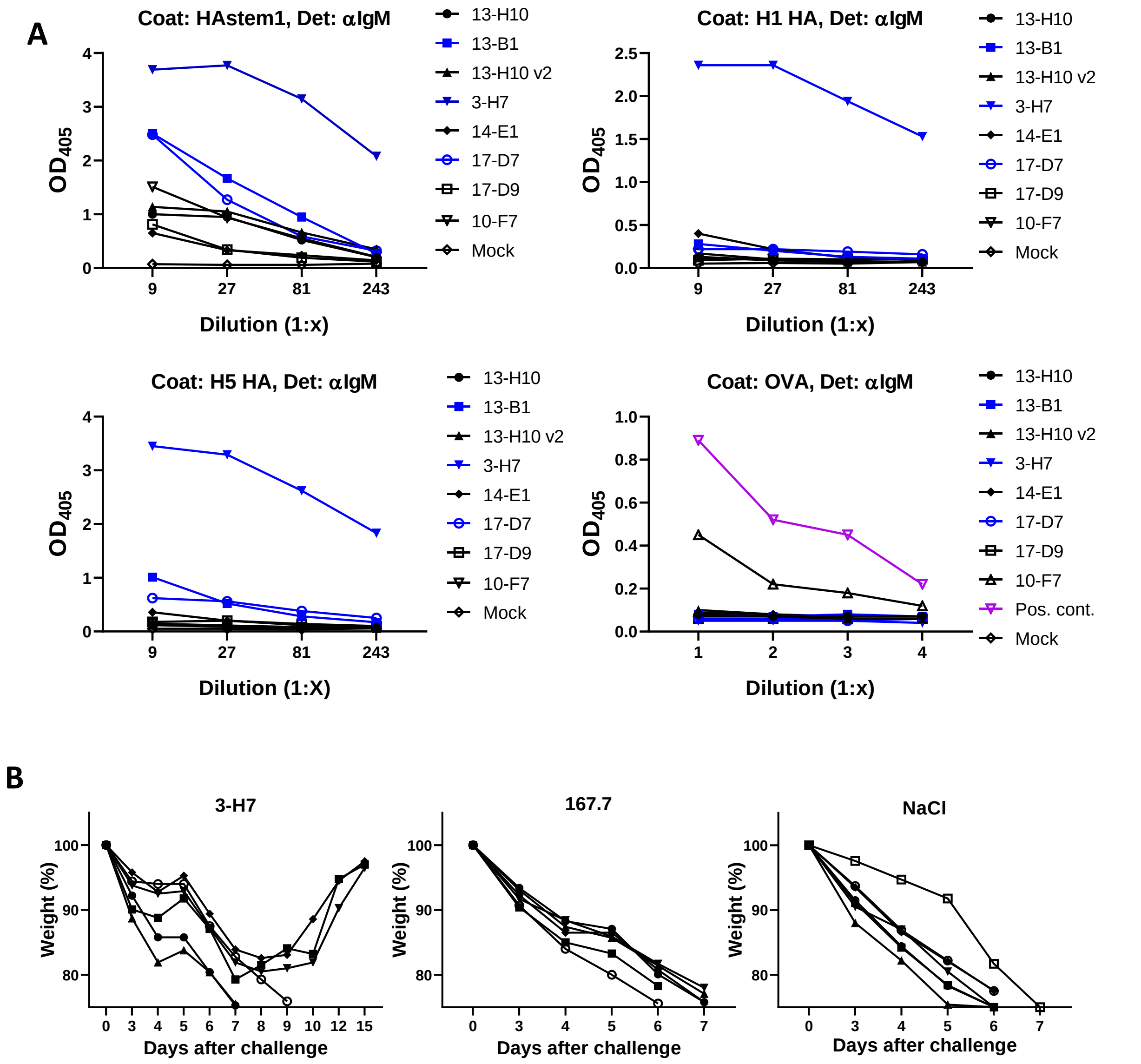

S3. ELISA of positive colonies and viral challenge after antibody transfer. (A) ELISA of positive colonies after fusion between splenocytes of mice immunized with αMHCII-H1stem and mouse plasmacytoma cells. Supernatants were assayed against the HA stem domain (coat with Phox-BSA and a recombinant protein with a Phox-specific scFv linked to the HA stem domain from influenza PR8), recombinant HA from PR8 (H1N1) and A/Hong Kong/483/97 (H5N1), and ovalbumin (OVA). (B) Weight of BALB/c mice that were treated with antibodies specific for the HA stem domain, obtained from hybridoma 3-H7 generated after vaccination with αMHCII-H1stem, or the isotype matched mAb 167.7, and challenged 24 hours later with a 5x lethal challenge of influenza PR8.

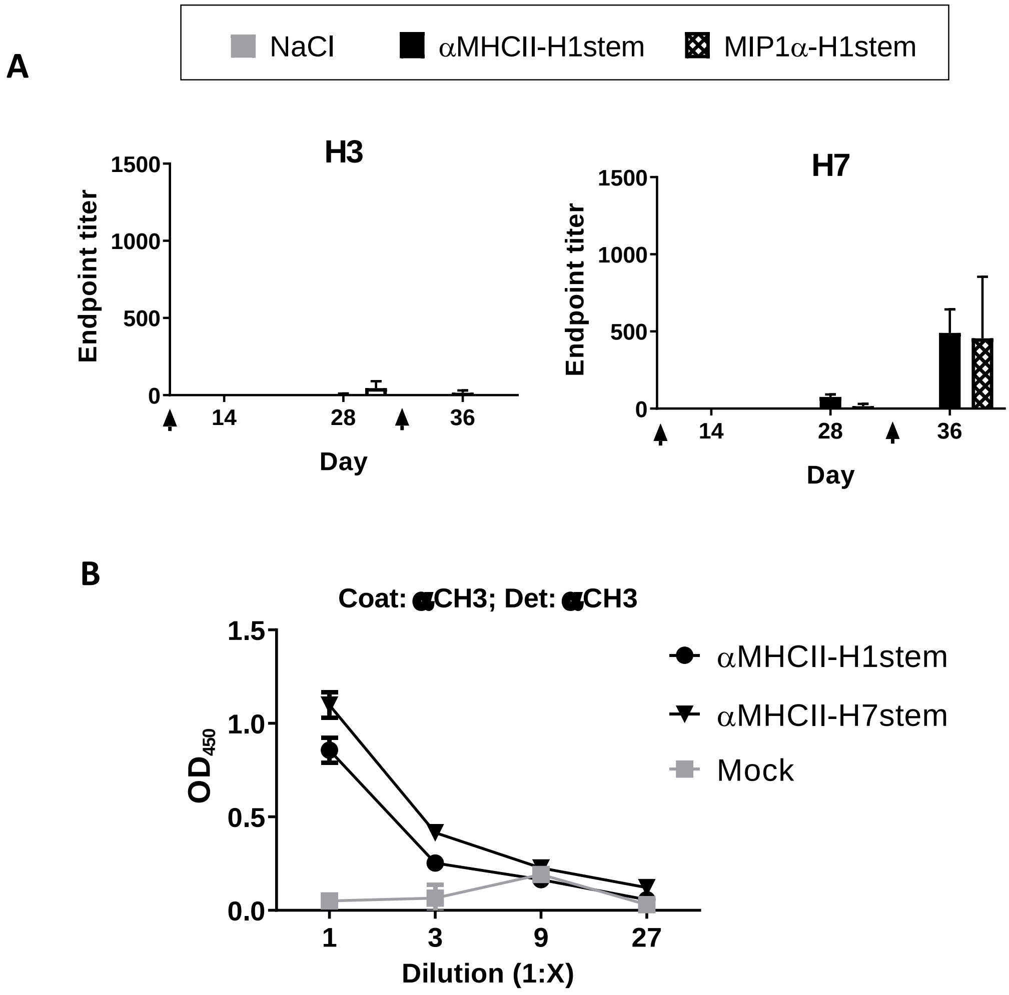

S4. Antibody responses after vaccination, and characterization of vaccine molecules. (A) BALB/c mice (n=10 mice/group) were immunized on days 0 and 29, as indicated by arrows, and sera analyzed for development of IgG responses against recombinant HA from A/Hong Kong/1/1968 (H3N2) and A/Shanghai/1/2013 (H7N9). Values given are mean +/- SEM. (B) Supernatants of transfected 293E cells were examined by ELISA using a mAb against C_H3 (dimerization unit) as coat. Proteins were detected with a biotinylated mAb against C_H3.
